# CD4^+^ T cell dysregulation in major depressive disorder is associated with sleep disturbance

**DOI:** 10.3389/fpsyt.2026.1763750

**Published:** 2026-01-29

**Authors:** Yao Gao, Zihan Lei, Yifan Ren, Xianyan Zhan, Zirong Chen, Zuer Guo, Ziyang Zhou, Xiancang Ma, Pan Li

**Affiliations:** 1 Department of Psychiatry, The First Affiliated Hospital of Xi’an Jiaotong University, Xi’an, China; 2 Center for Brain Science, The First Affiliated Hospital of Xi’an Jiaotong University, Xi’an, China; 3 The First Affiliated Hospital of Xi’an Jiaotong University, Shaanxi Provincial Key Laboratory of Biological Psychiatry, Xi’an, China; 4 Center for Translational Medicine, The First Affiliated Hospital of Xi’an Jiaotong University, Xi’an, China

**Keywords:** flow cytometry, inflammation, major depressive disorders, sleep disturbance, T cell

## Abstract

**Background:**

Emerging evidence supports the role of immune-mediated neuroinflammatory processes and disrupted sleep patterns in elevating susceptibility to major depressive disorder (MDD). Sleep disturbances, a hallmark clinical feature of MDD, have further been linked to changes in lymphocyte profiles. Nevertheless, the potential relationship between sleep disturbance and lymphocyte subpopulations characteristic in patients with MDD remains underexplored.

**Methods:**

In this study, flow cytometry was used to measure the proportion of peripheral blood CD4^+^ T-helper cells in 63 patients with MDD and 60 age- and sex-matched healthy controls (HCs). The relationship between self-reported sleep disturbances and the proportion of these cells was evaluated using Pearson’s correlation coefficient.

**Results:**

Baseline scores on the Hamilton Depression Rating Scale (HAMD) and Self-Rating Depression Scale (SDS) in patients with MDD were significantly higher than those in HCs. Regardless of antidepressant medication use, patients with MDD exhibited elevated proportions of CD4^+^ regulatory T cells (Tregs), IFN-γ^+^-Tregs, IL-4^+^-Tregs and Th1 (IFN-γ^+^-CD4^+^ T) cells compared to HCs. Furthermore, the Pittsburgh Sleep Quality Index (PSQI) scores in patients with MDD showed a positive correlation with CD4^+^ T cell frequency. Notably, MDD patients with self-reported sleep disturbance had a higher CD4^+^ T cell percentage than those without such disturbance.

**Conclusions:**

Our findings demonstrate that patients with MDD comorbid with sleep disturbances exhibit elevated proportions of CD4^+^ T cells compared to those without such disturbance. These results suggest that targeted interventions addressing sleep disruption may contribute to restoring CD4^+^ T cell homeostasis, potentially offering a novel therapeutic strategy for MDD management.

## 1 Introduction

Major depressive disorder (MDD), a prevalent neuropsychiatric condition affecting 10-20% of the global population, constitutes the leading cause of disability-adjusted life years worldwide (1–3). Despite decades of research into monoaminergic therapeutic, approximately one-third of patients exhibit treatment resistance to conventional antidepressants (4–6) such as selective serotonin reuptake inhibitors (SSRIs) and serotonin-norepinephrine reuptake inhibitors (SNRIs), underscoring critical deficiencies in our understanding of MDD pathogenesis. This has spurred the development of therapies acting on alternative neurobiological pathways. Notably, glutamatergic modulators, particularly the NMDA receptor antagonist esketamine, have emerged as a breakthrough for treatment-resistant depression (TRD). Their rapid antidepressant effects are hypothesized to involve downstream effects on neuroplasticity and inflammation (7, 8). Emerging translational paradigms now implicate systemic immunoinflammatory dysregulation as a pivotal mechanism (9, 10), with preclinical models demonstrating stress-induced microglial activation (11) and LPS-triggered neuroinflammatory cascades (12). At the core of this association lies dysregulated neuroimmune signaling, where peripheral inflammatory mediators access the brain, leading to microglial activation and altered neurocircuitry. This process can give rise to distinct cytokine-driven clinical phenotypes, often characterized by motivational deficits and cognitive impairment (13). Notably, these pathways are not disease-specific but represent a transdiagnostic pathophysiological process. Evidence from comorbidities shows that shared mechanisms, such as NF-κB signaling, BDNF/TrkB pathways, and HPA-axis dysregulation, underpin the relationship between depression and conditions ranging from psoriasis and atopic dermatitis to chronic viral infections like HIV/HBV (13, 14). Furthermore, environmental triggers like air pollutants can induce systemic inflammation, thereby engaging these shared pathways and contributing to depression risk (15), further solidifying the inflammation-depression link within a broader ecological framework. Clinical observations further substantiate this association, evidenced by elevated MDD incidence in IFN-α-treated hepatitis cohorts (16) and allergic diseases (asthma and rhinitis) (17, 18), paralleled by mechanistic findings of immunological deregulation involving inflammatory escalation (IL-6, TNF-α and CRP), monocytic TLR4/NF-κB hyperactivation (19–21), and cytotoxic NK cell deficiency (22), collectively delineating immunopathological profile of MDD.

The reciprocally potentiating relationship between sleep and MDD pathophysiology constitutes a clinically significant yet mechanistically elusive comorbidity, with polysomnographic studies positioning sleep architecture disturbances not merely as diagnostic epiphenomena but as transdiagnostic vulnerability factors (23). Meta-analytic syntheses reveal insomnia elevates MDD incidence risk (24, 25), while residual sleep fragmentation persists in pharmacologically remitted patients (26), is associated with an increase in the likelihood of recurrent depression over the subsequent year predicting (27). Our group’s prospective cohort investigations identified premonitory polysomnographic signatures of MDD pathogenesis: diminished sleep efficiency (SE ≤85%) and prolonged wake after sleep onset (WASO≥60 minutes) conferred 2.4-fold hazard ratios for incident depression (28), while NREM Stage 1 hyperdominance (>8% total sleep time) demonstrated significant predictive validity for depressive symptom emergence (adjusted OR = 3.17, p<0.001) (29). Notably, these sleep microstructure perturbations-encompassing REM latency prolongation and delta power reduction persist as treatment-refractory endophenotypes (30, 31), suggesting oscillatory dysregulation may transduce psychiatric vulnerability through cortico-limbic circuit destabilization (32, 33). This evidence collectively advocates for reconceptualizing sleep neurophysiology as both biomarker and biosystem modulator within the MDD pathogenesis to advance mechanism-targeted interventions.

Emerging evidence implicates sleep-mediated neuroimmune crosstalk in neuropsychiatric pathogenesis (34), with murine models demonstrating that sleep deprivation impairs glymphatic Aβ clearance (35, 36) and induces microglial priming-pathogenic processes implicated in dementia progression (37, 38). While immunoinflammatory axis dysregulation has been well-characterized in MDD pathogenesis, the specific immunological sequelae of comorbid sleep disturbances remain underexplored. Convergent findings identify sleep fragmentation as a potent immunomodulator: Human studies demonstrated that acute sleep restriction (4h/night × 5d) reduces natural killer (NK) cell cytotoxicity by 72% (*p* < 0.01) (39) and CD8^+^ T cell proliferation by 58% (*p* < 0.05) in healthy cohorts (40). Emerging evidence highlights the immunological interface between sleep disturbance and MDD pathophysiology. Notably, Hideo Suzuki et al. demonstrated that within the MDD cohort, self-reported sleep disturbances were significantly associated with increased effector memory CD8^+^ T cell prevalence concurrent with reduced CD56^+^ CD16^+^ natural killer cell populations (22). Complementing these findings, Dominique Piber et al. revealed that sleep fragmentation induces coordinated activation of cellular inflammatory markers and transcriptional aging signatures, potentially bridging sleep dysregulation with age-related comorbidities (41). These convergent mechanisms suggest lymphocyte dysfunction may mediate sleep-depression comorbidity. Targeted interventions could ameliorate sleep impairment through immunomodulatory pathways, while advancing sleep health may offer novel therapeutic opportunities for chronic infectious, inflammatory, and neuropsychiatric diseases (42).

Although Ke-qi Fan discovered that the absence of CD4^+^ T cells can protect mice from stress-induced anxiety-like behaviors, and physical stress can trigger severe mitochondrial fission in CD4^+^ T cells, subsequently leading to various behavioral abnormalities including anxiety, depression, and social disorders (43), the specific CD4^+^ T cell subset involved remains unclear. This study aims to characterize alterations in CD4^+^ T cell subsets in patients with MDD and to investigate the association between sleep disturbances and this immune dysregulation. We hypothesize that specific CD4^+^ T cell subsets are dysregulated in MDD and that this dysregulation is closely linked to sleep disturbances. To test this hypothesis, our primary aims are to comprehensively profile CD4^+^ T cell subsets in the peripheral blood of MDD patients compared to healthy controls using flow cytometry, and to assess the correlation between the severity of sleep disturbances and specific alterations in CD4^+^ T cell subsets. Our secondary aims include to preliminarily explore differences in CD4^+^ T cell profiles between antidepressant-treated and untreated MDD patients, and to provide preliminary evidence supporting sleep-focused immunomodulation as a potential therapeutic strategy for inflammatory MDD subtypes exhibiting limited response to conventional antidepressants. We anticipate that elucidating the interplay between sleep and CD4^+^ T cell immunity will help establish sleep-focused interventions as a promising therapeutic direction for inflammatory depression.

## 2 Materials and methods

### 2.1 Subjects

This single-center cross-sectional study was conducted at the Department of Psychiatry, The First Affiliated Hospital of Xi’an Jiaotong University. Acute-phase inpatients meeting DSM-5 diagnostic criteria for MDD were consecutively enrolled between September 2023 and February 2025. Exclusion criteria comprised: (1) other psychiatric disorders; (2) pregnant individuals; (3) acute infections, allergic reactions, or vaccinations within four weeks; (4) immunological conditions or immunomodulatory therapies; (5) untreated metabolic dysregulation. Sixty demographically matched healthy controls (HCs) were recruited from community populations, adhering to identical somatic exclusion protocols.

The study protocol obtained ethical clearance from the Institutional Review Board of The First Affiliated Hospital of Xi’an Jiaotong University (No. XJTU1AF2022LSK-002), with all procedures strictly compliant with the Declaration of Helsinki. Written informed consent was secured from all participants prior to study initiation.

### 2.2 Assessments

Comprehensive sociodemographic and clinical data were systematically collected from all participants, encompassing age, gender, body mass index (BMI), education duration, and familial psychiatric history of first/second-degree relatives with DSM-5-diagnosed mental disorders. Subjective sleep quality was evaluated across all subjects using the Pittsburgh Sleep Quality Index (PSQI) with a 30-day recall period, while depressive symptom profiles were concurrently assessed through the Self-Rating Depression Scale (SDS), capturing affective, cognitive, and somatic dimensions in both patients and HCs. For MDD patients specifically, licensed psychiatrists with ≥5 years of clinical specialization administered the 24-item Hamilton Depression Rating Scale (HAMD-24) to objectively quantify symptom severity.

### 2.3 Peripheral blood mononuclear cells isolating

Blood samples were collected from the elbow vein. 10 mL of fasting venous blood was drawn between 6:00 and 10:00 in the morning and placed into anticoagulation tubes. Peripheral blood mononuclear cells (PBMCs) were isolated from whole blood samples using density gradient centrifugation (400g, 30 minutes, acceleration rate 1, deceleration rate 0). The detailed procedure was as follows: The whole blood was diluted with 1× Dulbecco’s Phosphate-Buffered Saline (DPBS), at a ratio of 1:1. The diluted blood was then slowly added to the upper layer of an equal volume of Ficoll-Paque Plus (Cytiva, Sweden) using a Pasteur pipette. After centrifugation, the buffy coat layer (PBMC layer) in the middle was collected into a centrifuge tube. DPBS was added to the collected PBMCs, mixed well, and then centrifuged. This washing step was repeated twice. Finally, the PBMCs were collected by centrifugation and resuspended in 1 ml of cell cryopreservation solution (DONGXISW, Xi’an Shaanxi, China) and stored at -80°C.

### 2.4 Flow cytometry

PBMCs were thawed and resuspended in culture medium, then subjected to polyclonal stimulation using 50 ng/mL phorbol 12-myristate 13-acetate (PMA), 1 μg/mL ionomycin, and 1 × GolgiStop™ protein transport inhibitor (BD Biosciences, USA) under standard culture conditions (37°C, 5% CO_2_) for 4 hours to induce cytokine production. Viable cells were identified through 30-minute incubation with Zombie Aqua™ Fixable Viability Dye (BioLegend, USA), followed by sequential surface staining with anti-human CD4-FITC (clone OKT4), CD25-APC (clone BC96), and CD127-PE-Cy7 (clone A019D5) antibodies at 4°C protected from light. Cells were subsequently fixed and permeabilized using the BD Cytofix/Cytoperm™ Kit, enabling intracellular detection of IFN-γ-PE-Cy7 (clone B 27), IL-4-PE-Cy7 (clone MP4-25D2), and IL-17A-PE-Cy7 (clone BL168) through 45-minute incubation. All antibody incubations maintained strict light protection to prevent fluorochrome degradation. Flow cytometric acquisition was performed on a NovoCyte 3000RY instrument (Agilent, California, USA) calibrated daily with CST^®^ tracking beads, with compensation and population gating analyses executed in FlowJo™ v10.9.0 (Tree Star, Oregon, USA) using fluorescence-minus-one (FMO) controls for threshold determination.

### 2.5 Statistics analysis

Participants were categorized into three groups for analysis: patients with MDD who were drug-naïve (MDD-dn), patients with MDD who were drug-treated (MDD-dt), and HCs. The stratification of the MDD cohort into MDD-dn and MDD-dt subgroups was performed *a priori* to control for the potential confounding effects of psychotropic medications on immune parameters (44). This design allows for the examination of whether immune alterations are inherent to the pathophysiology of MDD or are influenced by medication status, thereby strengthening the interpretability of comparisons with HCs.

All data were analyzed using SPSS 25.0 (IBM Corp., Chicago, IL, USA) with a two-tailed significance threshold of *p* < 0.05. Three-group comparisons of demographic/clinical variables (age, sex, BMI, education, smoking history, HAMD-24/SDS/PSQI scores) were performed using one-way ANOVA followed by Bonferroni *post-hoc* tests. Immune cell percentages were analyzed through ANOVA for unadjusted comparisons and ANCOVA with covariate adjustment (age, sex, BMI, education). Bivariate Pearson correlations examined: 1) associations between global sleep disturbance (PSQI total/subdomain score) and both total/subdomain HAMD-24 scores; 2) relationships between PSQI scores and immune cell profiles in patients with MDD. Significant correlations were further verified via partial correlation analyses controlling for demographic confounders. To isolate sleep-specific effects, modified depression severity scores (HAMD-24/SDS with sleep items excluded) were calculated. Finally, independent t-tests compared immune cell percentages between patients with MDD with (PSQI > 10) and without (PSQI ≤ 10) clinically significant sleep disturbance.

## 3 Results

### 3.1 Demographic and clinical characteristics

**Figure 1:**
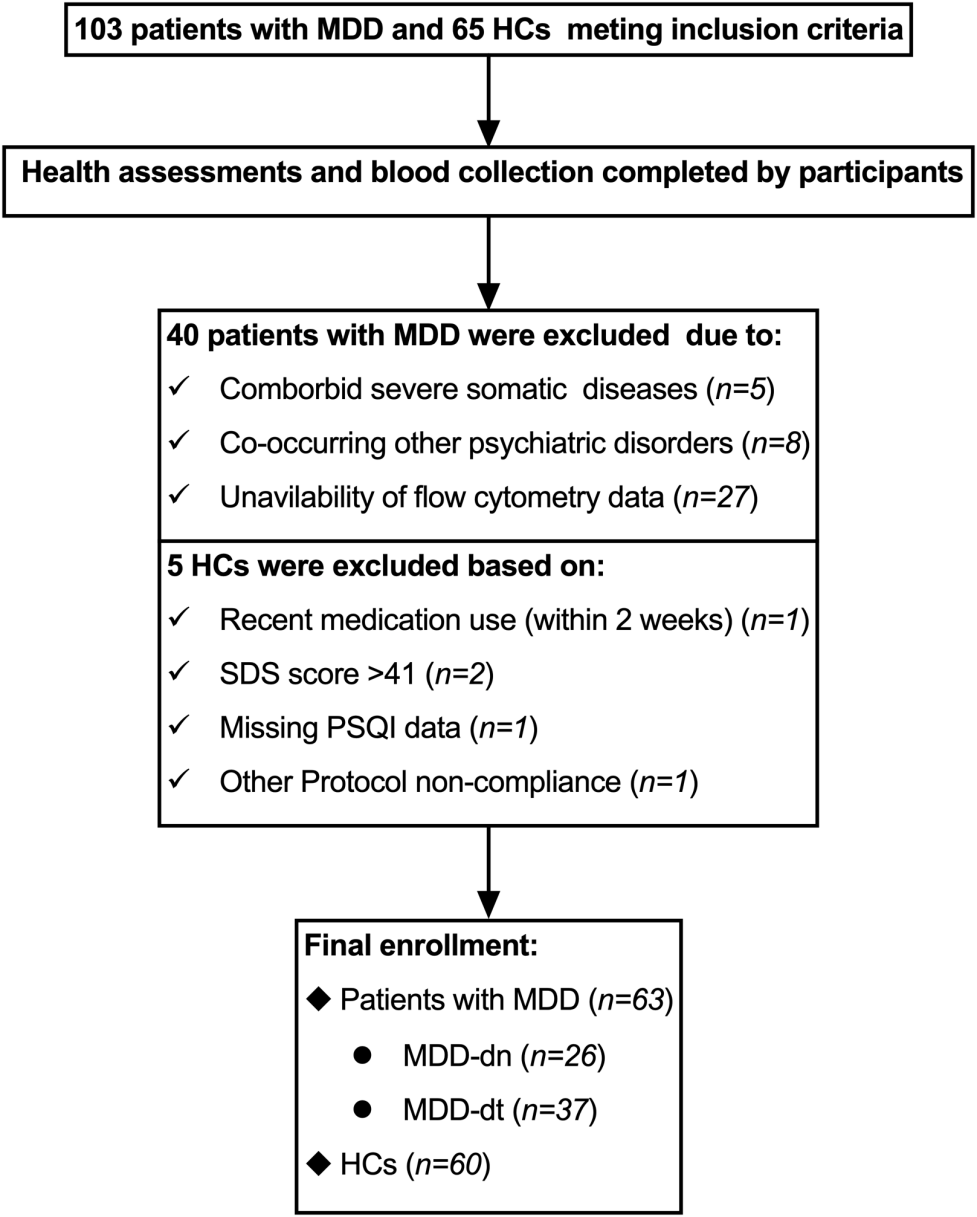
Flowchart of participants. Following rigorous onsite phenotyping of 168 initially screened candidates, 123 participants met stringent inclusion thresholds, constituting the final analytic cohort: 63 individuals diagnosed with MDD (26 drug-naïve [MDD-dn]; 37 drug-treated [MDD-dt]) and 60 demographically matched healthy controls (HCs). MDD, major depressive disorders; SDS, Self-Rating Depression Scale; PSQI, Pittsburgh Sleep Quality Index.

Among 168 potential participants screened, 123 met the inclusion criteria after on-site evaluation, including 63 patients with MDD (26 MDD-dn and 37 MDD-dt) and 60 HCs (**Figure 1**). Detailed medication histories from the preceding three months revealed: 41% (*n* = 26) were antidepressant-naïve; pharmacological interventions were administered to 59% (*n* = 37) of participants, with 59% of medicated patients receiving dual-class combination therapies (≥ 2 psychotropic categories) and 35% undergoing triple-class or more intensive regimens. Antidepressants predominated the treatment profiles (SSRIs 41%, SNRIs 19%, NRIs 22%), complemented by atypical antipsychotics (17%), benzodiazepines (32%), and non-benzodiazepine hypnotics (16%). The cohort included cases receiving adjunctive lithium (*n* = 2) and melatonin receptor agonists (*n* = 1), with medication records unavailable for 3% (*n* = 2) of participants.

The demographic and clinical characteristics of all participants are summarized in **Table 1**. No significant differences were observed between the MDD group and HCs in age, sex distribution, BMI, or smoking status (all *p*>0.05). However, significant differences were noted in educational attainment, family history of mental disorders, SDS scores, PSQI subscale scores, and HAMD-24 subscale scores (all *p* < 0.05). Of note, no statistically significant difference in emotional symptom scores was observed between the MDD-dn and MDD-dt subgroups (**Table 2**).

**Table 1:** Demographic and clinical characteristics of the participants.

Characteristics	HC (n=60)	MDD-dn (n=26)	MDD-dt (n=37)	*P*-value MDD-dn/HC	*P*-value MDD-dt/HC	*P*-value MDD-dn/MDD-dt
Age (years)	28.80 (8.03)	26.77 (8.73)	29.86 (10.09)	0.989	1.000	0.521
Sex						
Male, n (%)	24 (40.0)	8 (30.8)	11 (29.7)	0.474	0.386	1.000
Female, n (%)	36 (60.0)	18 (69.2)	26 (70.3)			
BMI (kg/m^2^)	22.44 (3.11)	22.61 (3.53)	23.54 (4.36)	0.988	0.440	0.116
Educational status						
≤Junior high school	12 (20.0)	1 (3.8)	3 (8.1)	**0.001**	**<0.001**	0.656
Senior high school	2 (3.3)	6 (23.1)	12 (32.4)			
College degree	27 (45.0)	17 (65.4)	21 (56.8)			
≥Master’s degree	19 (31.7)	2 (7.7)	1 (2.7)			
Smoking, n (%)	11 (18.3)	2 (7.7)	5 (13.5)	0.327	0.587	0.690
Duration of current episode (months)	–	5.64 (6.57)	4.65 (6.07)	–	–	–
Total episode (months)	–	34.35 (52.95)	61.51 (51.76)	–	–	–
Family history n (%)	0 (0.0)	3 (11.5)	5 (13.5)	**0.025**	**0.007**	1.000

Data are expressed as mean (SD) for continuous variables and count (percentage) for categorical variables, with group comparisons performed using one-way ANOVA (with Bonferroni-corrected *post hoc* tests for normally distributed variables confirmed by Shapiro-Wilk testing) or Pearson’s χ² test (with Fisher’s exact test for cells with expected frequencies <5), respectively. Any statistical parameter in bold met a significance level of 0.05. Abbreviations: SD (standard deviation), BMI (body mass index), HC (healthy controls), MDD-dn (drug-naïve major depressive disorder patients), MDD-dt (drug-treated major depressive disorder patients).

**Table 2:** Emotional and sleep assessments of the participants.

Characteristics	HCs (n=60)	MDD-dn (n=26)	MDD-dt (n=37)	*P*-value MDD-dn/HC	*P*-value MDD-dt/HC	*P*-value MDD-dn/MDD-dt
SDS	26.02 (4.71)	58.92 (9.03)	55.57 (8.78)	**<0.001**	**<0.001**	0.206
HAMD-24	0.32 (0.73)	22.42 (8.65)	23.76 (8.16)	**<0.001**	**<0.001**	1.000
HAMD-Anxiety/somatization	0.13 (0.39)	5.08 (2.83)	5.27 (2.50)	**<0.001**	**<0.001**	1.000
HAMD-Weight	0.00 (0.00)	0.58 (0.90)	0.49 (0.87)	**<0.001**	**0.001**	1.000
HAMD-Cognitive impairment	0.00 (0.00)	4.96 (3.17)	5.14 (2.71)	**<0.001**	**<0.001**	1.000
HAMD-Diurnal variation	0.00 (0.00)	0.85 (0.83)	0.54 (0.61)	**<0.001**	**<0.001**	0.059
HAMD-Retardation	0.00 (0.00)	8.42 (3.43)	8.65 (2.76)	**<0.001**	**<0.001**	1.000
HAMD-Sleep disturbance	0.17 (0.49)	3.69 (2.15)	4.00 (1.93)	**<0.001**	**<0.001**	1.000
HAMD-Feelings of despair	0.00 (0.00)	5.54 (3.10)	5.86 (2.36)	**<0.001**	**<0.001**	1.000
PSQI	4.12 (2.37)	10.38 (4.17)	11.59 (3.72)	**<0.001**	**<0.001**	0.444
A Subjective sleep quality	0.72 (0.61)	1.85 (0.73)	1.86 (0.82)	**<0.001**	**<0.001**	1.000
B Sleep latency	1.00 (0.99)	2.27 (0.78)	2.00 (0.94)	**<0.001**	**<0.001**	0.790
C Sleep duration	0.63 (0.61)	1.62 (1.24)	1.30 (1.23)	**<0.001**	**0.003**	0.564
D Sleep efficiency	0.38 (0.56)	0.85 (0.83)	0.92 (0.95)	**0.030**	**0.003**	1.000
E Sleep disturbances	0.43 (0.50)	1.50 (0.71)	1.46 (0.69)	**<0.001**	**<0.001**	1.000
F Use of sleep medication	0.07 (0.36)	1.31 (1.41)	1.81 (1.43)	**<0.001**	**<0.001**	0.187
G Daytime dysfunction	0.35 (0.55)	1.27 (0.72)	1.41 (0.80)	**<0.001**	**<0.001**	1.000

Data are expressed as mean (SD) for continuous variables with group comparisons performed using one-way ANOVA (with Bonferroni-corrected *post hoc* tests for normally distributed variables confirmed by Shapiro-Wilk testing). Any statistical parameter in bold met a significance level of 0.05. Abbreviations: SD (standard deviation), HC (healthy controls), MDD-dn (drug-naïve major depressive disorder patients), MDD-dt (drug-treated major depressive disorder patients), HAMD-24 (24-item Hamilton Rating Scale for Depression), SDS (Self-Rating Depression Scale), PSQI (Pittsburgh Sleep Quality Index).

### 3.2 Dysregulated CD4^+^ T cell subpopulation distribution in patients with MDD

Both MDD-dn and MDD-dt patients demonstrated significantly elevated frequencies of Treg cells in peripheral blood compared to HCs, even after accounting for age, sex, BMI, and educational status (**Table 3**). In the MDD-dn subgroup, T helper 1 (Th1, IFN-γ^+^-CD4^+^ T) cells, IFN-γ^+^-Tregs, and IL-4^+^-Tregs exhibited significantly higher proportions than HCs in covariate-adjusted analyses. Notably, no intergroup differences were detected between MDD-dn and MDD-dt patients for these immune markers. Furthermore, CD4^+^ T cell populations, T helper 2 (Th2, IL-4^+^-CD4^+^ T) cells, T helper 17 (Th17, IL-17A^+^-CD4^+^ T) cells, and IL-17A^+^-Tregs remained statistically comparable across all study groups (**Table 3**).

**Table 3:** Summary of comparisons in the percentage of live peripheral blood mononuclear cells (PBMCs) type of the participants (n=123).

Cell subpopulation	MDD-dn (n=26)	MDD-dt (n=37)	HC (n=60)	*P*-value MDD-dn/HC	*P*-value MDD-dt/HC	*P*-value MDD-dn/MDD-dt
ANOVA						
CD4^+^	39.91 (2.05)	39.56 (1.91)	37.90 (0.90)	1.000	1.000	1.000
Treg	6.80 (0.58)	6.39 (0.35)	4.94 (0.29)	**0.003**	**0.012**	1.000
IFN-γ^+^-Treg	4.41 (0.50)	4.33 (0.50)	3.02 (0.29)	**0.071**	**0.046**	0.905
IL-4^+^-Treg	2.47 (0.41)	1.90 (0.26)	1.42 (0.16)	**0.005**	0.142	0.162
IL-17A^+^-Treg	1.36 (0.67)	1.49 (0.56)	1.57 (0.60)	0.374	0.746	0.639
Th1	20.93 (1.92)	18.90 (1.85)	15.86 (1.40)	0.143	0.539	1.000
Th2	3.33 (0.52)	2.25(0.32)	2.19 (0.40)	0.236	1.000	0.374
Th17	1.65 (0.17)	1.68 (0.16)	1.53 (0.20)	1.000	1.000	1.000
ANCOVA1						
CD4^+^	39.90 (1.84)	39.61 (1.56)	37.87 (1.22)	1.000	1.000	1.000
Treg	6.79 (0.46)	6.50 (0.38)	4.87 (0.30)	**0.002**	**0.003**	1.000
IFN-γ^+^-Treg	4.40 (0.50)	4.40 (0.40)	3.00 (0.30)	**0.068**	**0.041**	1.000
IL-4^+^-Treg	2.46 (0.31)	1.93 (0.26)	1.40 (0.20)	**0.013**	0.350	0.560
IL-17A^+^-Treg	1.40 (0.20)	1.50 (0.20)	1.60 (0.20)	1.000	1.000	1.000
Th1	20.90 (2.10)	18.90 (1.80)	15.90 (1.40)	0.149	0.584	1.000
Th2	3.32 (0.53)	2.38 (0.45)	2.12 (0.35)	0.174	1.000	0.529
Th17	1.60 (0.20)	1.60 (0.30)	1.60 (0.20)	1.000	1.000	1.000
ANCOVA2						
CD4^+^	40.02 (1.74)	39.02 (1.47)	38.18 (1.14)	1.000	1.000	1.000
Treg	6.87 (0.45)	6.44 (0.39)	4.88 (0.30)	**0.001**	**0.006**	1.000
IFN-γ^+^-Treg	4.60 (0.50)	4.20 (0.40)	3.00 (0.30)	**0.032**	0.078	0.905
IL-4^+^-Treg	2.44 (0.31)	1.89 (0.27)	1.44 (0.20)	**0.023**	0.563	0.536
IL-17A^+^-Treg	1.40 (0.20)	1.30 (0.30)	1.60 (0.20)	0.934	1.000	1.000
Th1	21.70 (2.00)	18.50 (1.70)	15.80 (1.30)	**0.047**	0.657	0.692
Th2	3.30 (0.53)	2.38 (0.45)	2.13 (0.35)	0.212	1.000	0.588
Th17	1.60 (0.20)	1.50 (0.30)	1.60 (0.20)	1.000	1.000	1.000

Data are expressed as mean (SD), and values under MDD and HC represent the percentages (%) of cell types in live PBMCs. ANCOVA1 controlled for educational status, and ANCOVA2 controlled for age, sex, body mass index, and educational status. All *p*-values in this table are corrected by Bonferroni. Any statistical parameter in bold met a significance level of 0.05. Abbreviations: SD (standard deviation), HC (healthy controls), MDD-dn (drug-naïve major depressive disorder patients), MDD-dt (drug-treated major depressive disorder patients), Th (T helper), Treg (regulatory T).

### 3.3 Correlation of sleep quality and depression severity in patients with MDD

**Figure 2:**
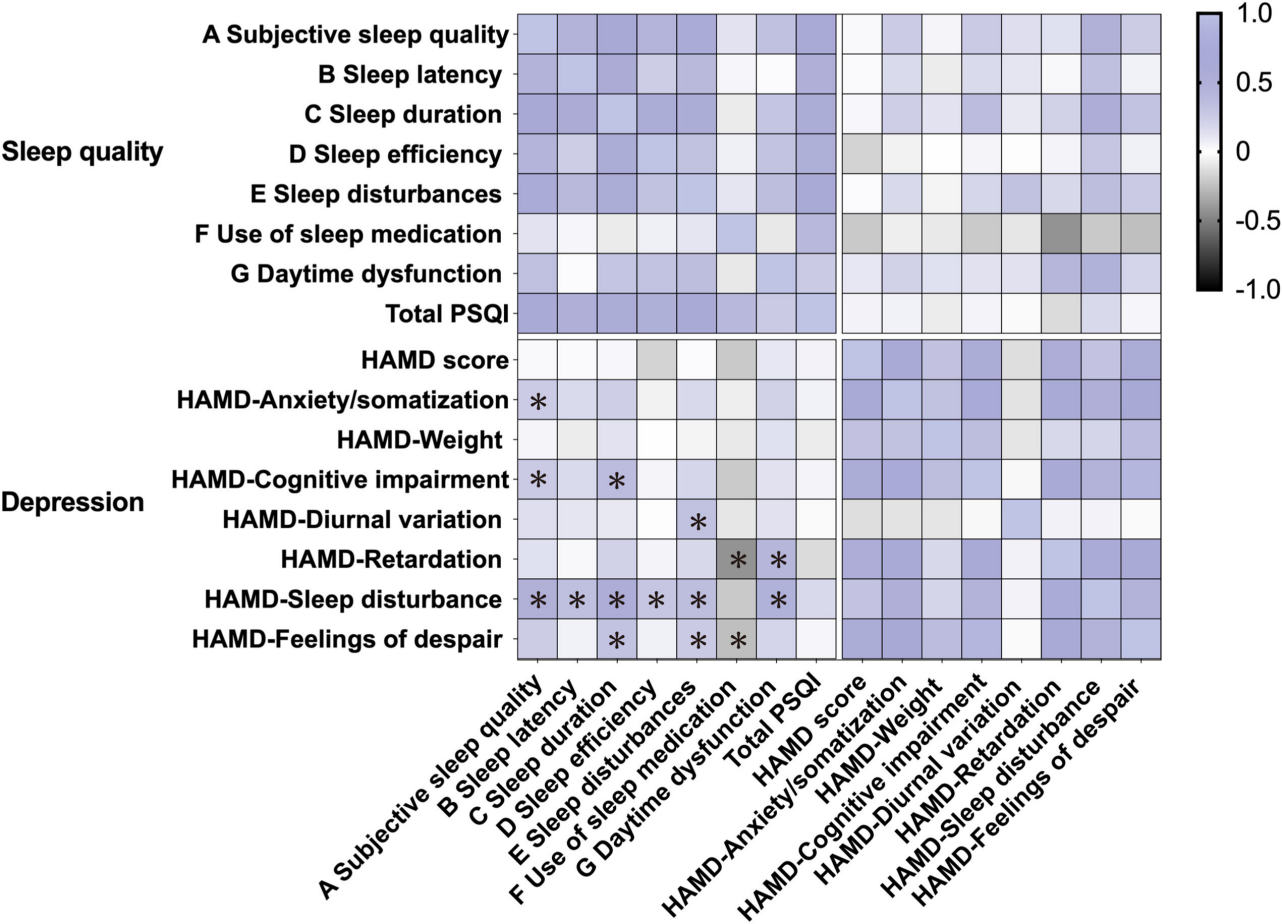
Correlation between sleep disturbance and depressive disorder in patients with MDD. Heatmap displays Pearson correlation coefficients between specific sleep parameters (PSQI components) and depression severity markers (HAMD-24 subscales) in patients with MDD (n=63). Color gradient indicates correlation strength (purple: positive correlation; black: negative correlation). *p < 0.05.

No significant association was observed between total PSQI and HAMD-24 scores (*p*>0.05). Subscale analyses revealed HAMD-sleep disturbance consistently correlated with four PSQI domains: prolonged latency (*r* = 0.335), shortened duration (*r* = 0.542), reduced efficiency (*r* = 0.286), and self-reported disturbance (*r* = 0.357) (all *p* < 0.05), with minimal attenuation in partial correlations. PSQI sleep disturbance additionally linked to HAMD-diurnal variation (*r* = 0.324) and despair (*r* = 0.262). Subjective sleep quality associated with anxiety/somatization (*r* = 0.25) and cognitive impairment (*r* = 0.262), though partial correlations weakened significance. Notably, sleep medication inversely predicted psychomotor retardation (*r* = -0.424) and despair (*r* = -0.252), whereas daytime dysfunction strongly correlated with sleep disturbance (*r* = 0.469) and psychomotor deficits (*r* = 0.416) (all *p* < 0.05, partial results consistent; **Figure 2**).

### 3.4 Correlation of sleep quality and immune cell profile in patients with MDD

**Figure 3:**
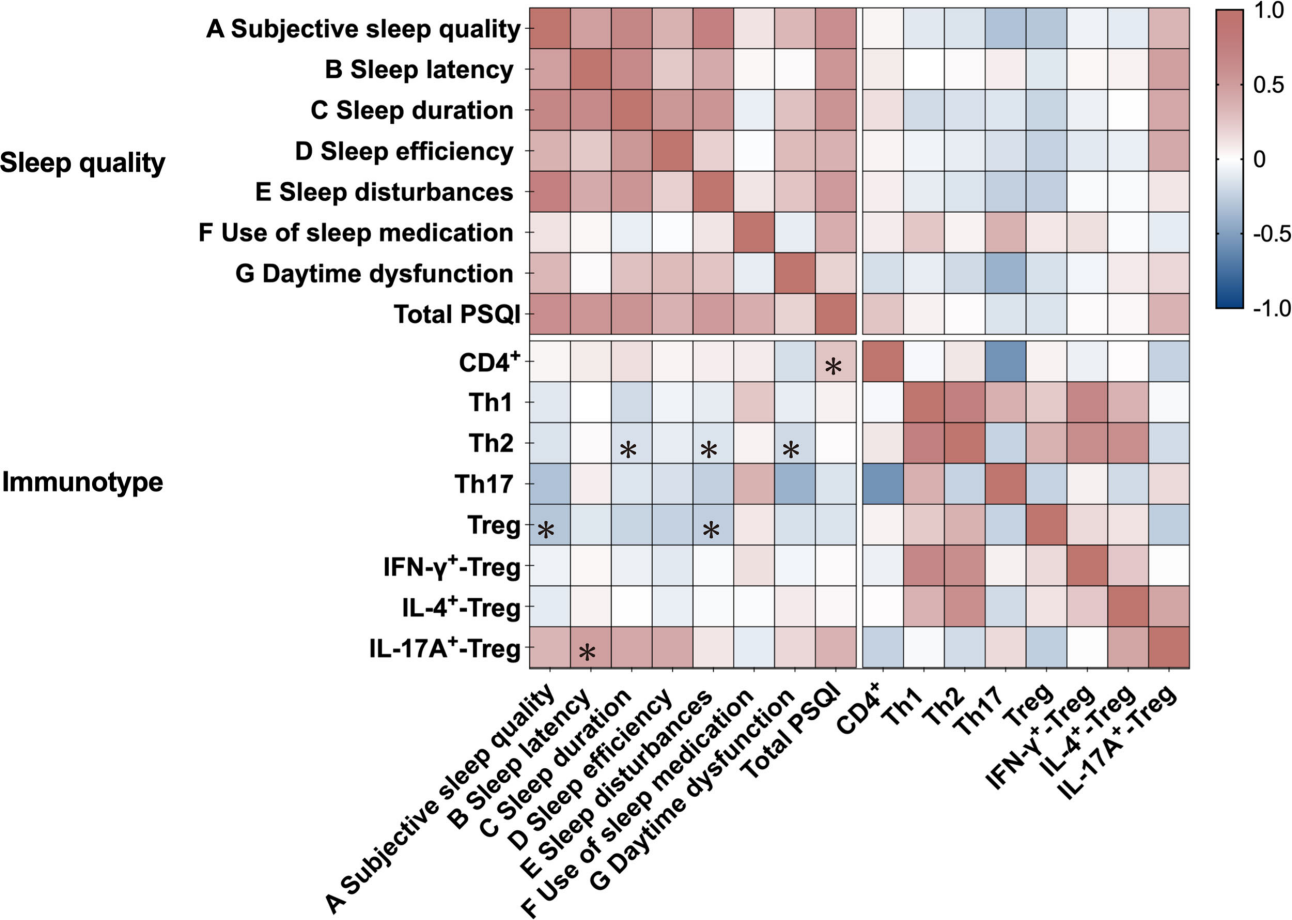
Correlation between sleep disturbance and immune cell profiles in patients with MDD. Heatmap displays Pearson correlation coefficients between specific sleep parameters (PSQI components) and peripheral immune cell subsets in patients with MDD (n=63). Color gradient indicates correlation strength (red: positive correlation; blue: negative correlation). *p < 0.05.

**Figure 4:**
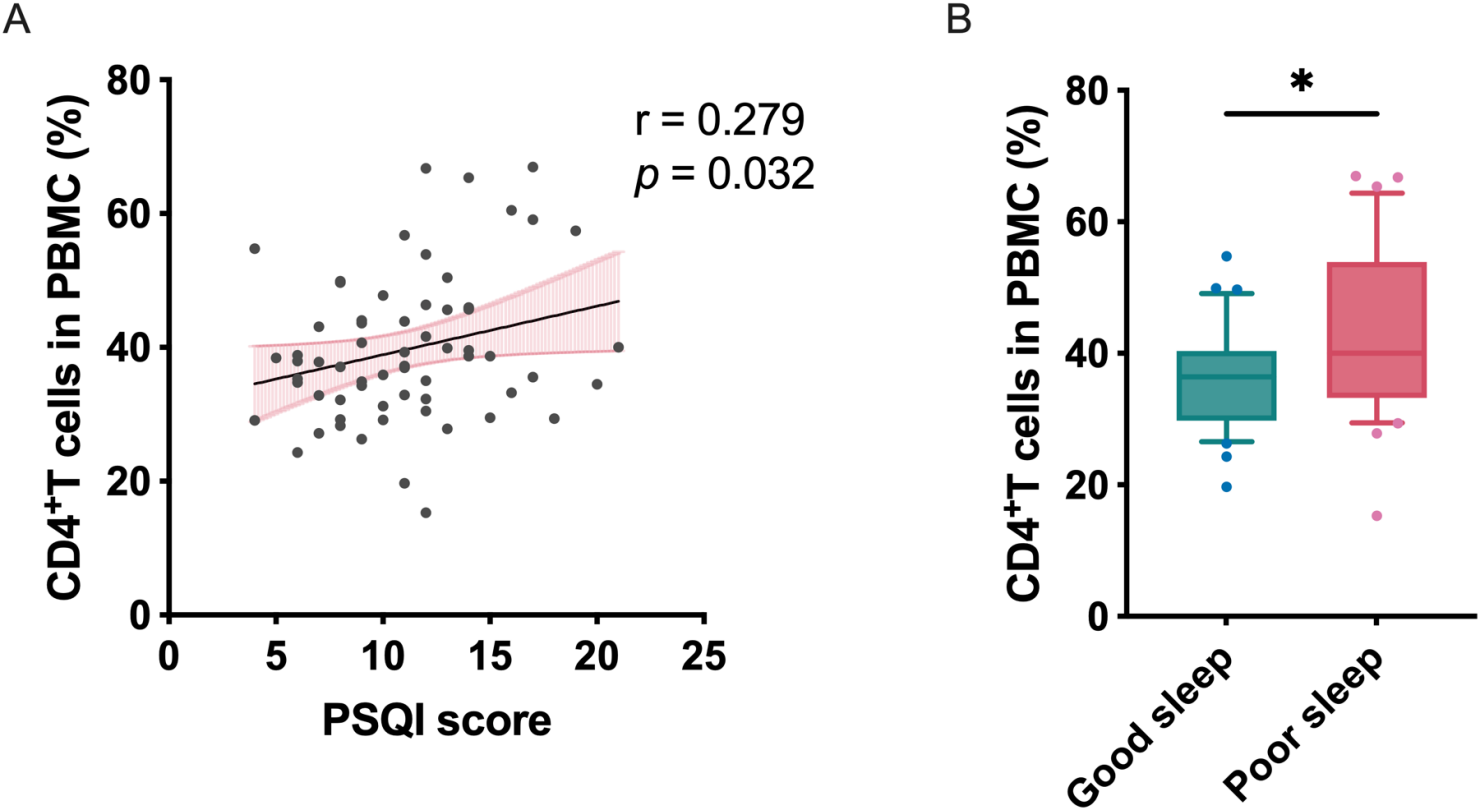
Sleep disturbance-associated CD4^+^ T cell alterations in major depressive disorder. **(A)** Correlation between PSQI score and peripheral CD4^+^ T cell percentage in patients with MDD; the correlation analysis was performed using Pearson’s correlation coefficients. **(B)** Increased CD4^+^ T cells proportion in MDD patients with sleep disorders compared to those without. Statistical significance was assessed by unpaired two-tailed Student’s t-test without multiple comparison adjustments. Values shown as mean ± standard error of the mean (SEM). *p < 0.05.

The total PSQI score was positively associated with CD4^+^ T cell frequency in patients with MDD, both in unadjusted (Pearson *r* = 0.258, *p* = 0.041) and depression severity-adjusted analyses (partial *r* = 0.279, *p* = 0.032) (**Figure 3**). Consistently, MDD patients with self-reported sleep disturbances exhibited higher CD4^+^ T cell levels compared to those without sleep disturbances, with significant differences in both unadjusted (*T* = 2.043, *p* = 0.046) and fully adjusted models (*F* = 2.389, *p* = 0.033; covariates: age, sex, BMI, education) (**Figure 4**). Subscale analyses revealed immune specificity: subjective sleep quality and sleep disturbances inversely correlated with Tregs frequency (*r* = -0.280 to -0.253, *p* = 0.038–0.053), while sleep latency positively associated with IL-17A^+^-Tregs (*r* = 0.481, *p* = 0.037; partial *r* = 0.511, *p* = 0.036). Daytime dysfunction and sleep duration showed negative associations with Th2 cells in adjusted models (partial *r* = -0.324 to -0.227, *p* = 0.012–0.034). A non-significant trend suggested elevated IL-4^+^-Tregs in MDD patients with sleep disturbances versus those without (*F*_1,61_ = 3.43, *p* = 0.096) after adjusting for depression severity. No global PSQI-Treg correlation was observed, highlighting the importance of symptom-level analyses.

## 4 Discussion

The primary objective of this study was to characterize the landscape of CD4^+^ T cell dysregulation in MDD and to investigate its relationship with the highly prevalent comorbidity of sleep disturbance. Our central finding is that patients with MDD exhibit a specific pattern of CD4^+^ T cell subset imbalance, independent of antidepressant exposure. Specifically, patients exhibited elevated frequencies of Tregs and effector Treg subsets (IFN-γ^+^-Tregs and IL-4^+^-Tregs) compared to HCs, regardless of pharmacological treatment status. While this aligns with recent reports of increased Treg proportions in depression cohorts (22), it contrasts with studies showing Treg depletion in MDD (45). This discrepancy may arise from methodological differences: prior work predominantly analyzed total Treg populations, whereas our subset-specific approach uncovered compensatory expansion of effector Tregs-a phenomenon observed in chronic inflammatory conditions such as cancer and vitiligo, where dysfunctional Tregs undergo pathogenic reprogramming into effector-like cells (46, 47). We hypothesize that the elevated effector Treg frequencies observed here may reflect a failed compensatory response to persistent neuroinflammation, akin to the exhausted T cell phenotypes seen in prolonged antigen exposure. Notably, the co-elevation of Th1 cells and IFN-γ^+^-Tregs in MDD-dn patients mirrors findings from interferon-driven rodent models of depression and clinical observations of interferon-α-induced depressive symptoms in hepatitis patients (16, 48). This parallelism suggests unresolved IFN-γ-mediated neuroinflammatory cascades may underpin specific MDD subtypes (49). Unexpectedly, the absence of Th17 cell alterations align with recent meta-analytic evidence (50), yet contradicts the microglial hyperactivation hypothesis, which posits IL-17A-driven neurotoxicity in depression (51). This divergence may stem from our focus on circulating lymphocytes rather than CNS-infiltrating or gut-resident Th17 populations, which exhibit distinct functional properties in neuroinflammation.

The PSQI emerges as a multidimensional tool capturing distinct neuropsychiatric pathways in MDD, validated by its high insomnia diagnostic accuracy (sensitivity: 98.7%; specificity: 84.4%) (52) and responsiveness to therapeutic improvements (53). While global PSQI-HAMD-24 score associations remained non-significant, consistent with studies of heterogeneous depression cohorts-our subscale stratification revealed specific correlations that suggest three potential mechanistic pathways linking sleep architecture to psychopathology: 1) Our data showed that subjective circadian disruption (reflected in PSQI subscales for sleep latency, duration, and efficiency) was significantly correlated with clinician-rated sleep disturbances on the HAMD-24 (r=0.335-0.542). This finding is consistent with neuroimaging studies linking similar sleep parameters to prefrontal GABA/glutamate imbalances (54); 2) We found that impairments in perceived sleep quality (PSQI) were correlated with both cognitive deficits (r=0.262) and psychomotor retardation (r=0.416) on the HAMD-24, a pattern that parallels the sleep fragmentation effects observed in conditions like Alzheimer’s disease (55); 3) Our results indicated a correlation between daytime dysfunction due to sleepiness and ratings of suicidal despair (r=0.469), which aligns with previously identified polysomnographic predictors of suicide risk (56). These pathways corroborate our prior findings on sleep stage-specific depression risks while extending the clinical relevance of PSQI subscales beyond insomnia screening to include cognitive preservation and suicide prevention strategies.

This study observed an inverse correlation between anti-inflammatory cells (Tregs and Th2) and sleep disturbances (57), while IL-17A^+^-Tregs within CD4^+^ T cells showed a positive correlation with sleep impairment. These findings may be associated with the broad immunological effects of disrupted sleep: previous studies have demonstrated that short or fragmented sleep increases the risks of type 2 diabetes (58), upper respiratory infections (59), cardiovascular diseases (60, 61), breast cancer (62), and all-cause mortality (63), with some pathologies partially linked to immune dysregulation (64). However, the precise mechanisms remain controversial (64, 65). Notably, although the overall Treg proportion was elevated in patients with MDD compared to HCs (*p* < 0.05), the subgroup with comorbid sleep disturbances exhibited insufficient anti-inflammatory compensation by Tregs, accompanied by increased pro-inflammatory subsets (e.g., IL-17A^+^-Tregs). This suggests that sleep impairment may undermine the functional compensatory capacity of Tregs, exacerbating inflammatory imbalance and depressive symptoms. These findings provide novel clinical evidence for understanding the role of sleep-related immune dysregulation in MDD progression. Our findings, which link specific sleep disturbances to CD4^+^ T cell dysregulation, support the recognition of an inflammation associated subtype of MDD. This subtype, often characterized by increased biomarkers like CRP, is consistently linked to poorer response to conventional antidepressant medications. Therefore, the sleep-immune profile observed in our study may serve as a practical clinical indicator. Assessing sleep architecture and immune markers could help identify patients who are less likely to benefit from conventional antidepressants. For these individuals, clinicians might consider alternative treatment strategies from the outset. Such strategies could include anti-inflammatory approaches or interventions with documented efficacy in TRD, such as electroconvulsive therapy (ECT) or ketamine, which are thought to work through mechanisms that may directly or indirectly modulate immune pathways. This approach underscores the potential of using sleep and immune measures to personalize treatment selection in MDD (66, 67).

### 4.1 Limitations

(1) Cross-sectional design precludes causal inference between sleep disturbance and immune shifts; (2) Lack of cytokine secretion capacity measurements limits functional interpretation of T cell frequencies; (3) The modest sample size necessitates replication in pharmacologically naïve cohorts. Future studies should employ actigraphy-verified sleep assessments and single-cell transcriptomics to resolve whether observed immune changes reflect peripheral recruitment or *in situ* differentiation; (4) Our assessment of sleep disturbances relied solely on the self-reported PSQI. While the PSQI is a well-validated instrument, it is subjective and lacks the objective validation provided by actigraphy or polysomnography. Future studies incorporating such objective measures are essential to corroborate our findings and more precisely quantify the relationship between sleep architecture and immune parameters; (5) For the MDD-dt subgroup, the heterogeneity in pharmacological regimens (including different classes of antidepressants, varying dosages, and treatment durations) represents a potential confounding factor that we could not fully control for in our analyses. Although stratifying the cohort into drug-naïve and drug-treated groups was a necessary first step, the differential immunomodulatory effects of specific medications may have influenced our results and contributed to the variability within the MDD-dt group. Future research with larger, pharmacologically homogeneous samples is needed to disentangle the specific effects of individual drugs on the immune system.

### 4.2 Conclusions

In summary, our findings provide important novel insights into the immunological pathways preferentially affected in MDD. Specifically, we have revealed an elevation of circulating CD4^+^ T cells in MDD patients with sleep disorder and demonstrated that this CD4^+^ T cells hyperactivation may be influenced by sleep disruptions. Finally, our results highlight the potential significance of CD4^+^ T cell overabundance in MDD pathophysiology. Further research employing experimental and longitudinal designs is warranted to elucidate the mechanisms underlying these immune abnormalities.

## Data Availability

The raw data supporting the conclusions of this article will be made available by the authors, without undue reservation.

## References

[1] Marx W Penninx B Solmi M Furukawa TA Firth J Carvalho AF . Major depressive disorder. Nat Rev Dis Primers. (2023) 9:44. doi: 10.1038/s41572-023-00454-1, PMID: 37620370

[2] Malhi GS Mann JJ . Depression. Lancet. (2018) 392:2299–312. doi: 10.1016/s0140-6736(18)31948-2, PMID: 30396512

[3] McCarron RM Shapiro B Rawles J Luo J . Depression. Ann Intern Med. (2021) 174:Itc65–itc80. doi: 10.7326/aitc202105180, PMID: 33971098

[4] Kishi T Ikuta T Sakuma K Okuya M Hatano M Matsuda Y . Antidepressants for the treatment of adults with major depressive disorder in the maintenance phase: a systematic review and network meta-analysis. Mol Psychiatry. (2023) 28:402–9. doi: 10.1038/s41380-022-01824-z, PMID: 36253442 PMC9812779

[5] Davey CG Chanen AM Hetrick SE Cotton SM Ratheesh A Amminger GP . The addition of fluoxetine to cognitive behavioural therapy for youth depression (YoDA-C): a randomised, double-blind, placebo-controlled, multicentre clinical trial. Lancet Psychiatry. (2019) 6:735–44. doi: 10.1016/s2215-0366(19)30215-9, PMID: 31371212

[6] Meltzer-Brody S Colquhoun H Riesenberg R Epperson CN Deligiannidis KM Rubinow DR . Brexanolone injection in post-partum depression: two multicentre, double-blind, randomised, placebo-controlled, phase 3 trials. Lancet. (2018) 392:1058–70. doi: 10.1016/s0140-6736(18)31551-4, PMID: 30177236

[7] Krystal JH Kavalali ET Monteggia LM . Ketamine and rapid antidepressant action: new treatments and novel synaptic signaling mechanisms. Neuropsychopharmacology. (2024) 49:41–50. doi: 10.1038/s41386-023-01629-w, PMID: 37488280 PMC10700627

[8] Chen H Zhao X Ma X Ma H Zhou C Zhang Y . Effects of esketamine and fluoxetine on depression-like behaviors in chronic variable stress: a role of plasma inflammatory factors. Front Psychiatry. (2024) 15:1388946. doi: 10.3389/fpsyt.2024.1388946, PMID: 38812484 PMC11133692

[9] McIntyre RS Subramaniapillai M Lee Y Pan Z Carmona NE Shekotikhina M . Efficacy of adjunctive infliximab vs placebo in the treatment of adults with bipolar I/II depression: A randomized clinical trial. JAMA Psychiatry. (2019) 76:783–90. doi: 10.1001/jamapsychiatry.2019.0779, PMID: 31066887 PMC6506894

[10] Patil CR Suryakant Gawli C Bhatt S . Targeting inflammatory pathways for treatment of the major depressive disorder. Drug Discov Today. (2023) 28:103697. doi: 10.1016/j.drudis.2023.103697, PMID: 37422168

[11] Biltz RG Sawicki CM Sheridan JF Godbout JP . The neuroimmunology of social-stress-induced sensitization. Nat Immunol. (2022) 23:1527–35. doi: 10.1038/s41590-022-01321-z, PMID: 36369271 PMC10000282

[12] Zhuang X Zhan B Jia Y Li C Wu N Zhao M . IL-33 in the basolateral amygdala integrates neuroinflammation into anxiogenic circuits via modulating BDNF expression. Brain Behav Immun. (2022) 102:98–109. doi: 10.1016/j.bbi.2022.02.019, PMID: 35181439

[13] Fabrazzo M Cipolla S Signoriello S Camerlengo A Calabrese G Giordano GM . A systematic review on shared biological mechanisms of depression and anxiety in comorbidity with psoriasis, atopic dermatitis, and hidradenitis suppurativa. Eur Psychiatry. (2021) 64:e71. doi: 10.1192/j.eurpsy.2021.2249, PMID: 34819201 PMC8668448

[14] Fabrazzo M Cipolla S Pisaturo M Camerlengo A Bucci P Pezzella P . Bidirectional relationship between HIV/HBV infection and comorbid depression and/or anxiety: A systematic review on shared biological mechanisms. J Pers Med. (2023) 13:1689. doi: 10.3390/jpm13121689, PMID: 38138916 PMC10744606

[15] Catapano P Luciano M Cipolla S D’Amico D Cirino A Della Corte MC . What is the relationship between exposure to environmental pollutants and severe mental disorders? A systematic review on shared biological pathways. Brain Behav Immun Health. (2025) 43:100922. doi: 10.1016/j.bbih.2024.100922, PMID: 39803412 PMC11719278

[16] Hauser P Khosla J Aurora H Laurin J Kling MA Hill J . A prospective study of the incidence and open-label treatment of interferon-induced major depressive disorder in patients with hepatitis C. Mol Psychiatry. (2002) 7:942–7. doi: 10.1038/sj.mp.4001119, PMID: 12399946

[17] Postolache TT Komarow H Tonelli LH . Allergy: a risk factor for suicide? Curr Treat Opt Neurol. (2008) 10:363–76. doi: 10.1007/s11940-008-0039-4, PMID: 18782509 PMC2592251

[18] Fang BJ Tonelli LH Soriano JJ Postolache TT . Disturbed sleep: linking allergic rhinitis, mood and suicidal behavior. Front Biosci (Schol Ed). (2010) 2:30–46. doi: 10.2741/s44, PMID: 20036927

[19] Miller AH Maletic V Raison CL . Inflammation and its discontents: the role of cytokines in the pathophysiology of major depression. Biol Psychiatry. (2009) 65:732–41. doi: 10.1016/j.biopsych.2008.11.029, PMID: 19150053 PMC2680424

[20] Slavich GM Irwin MR . From stress to inflammation and major depressive disorder: a social signal transduction theory of depression. Psychol Bull. (2014) 140:774–815. doi: 10.1037/a0035302, PMID: 24417575 PMC4006295

[21] Irwin MR Wang M Campomayor CO Collado-Hidalgo A Cole S . Sleep deprivation and activation of morning levels of cellular and genomic markers of inflammation. Arch Intern Med. (2006) 166:1756–62. doi: 10.1001/archinte.166.16.1756, PMID: 16983055

[22] Suzuki H Savitz J Kent Teague T Gandhapudi SK Tan C Misaki M . Altered populations of natural killer cells, cytotoxic T lymphocytes, and regulatory T cells in major depressive disorder: Association with sleep disturbance. Brain Behav Immun. (2017) 66:193–200. doi: 10.1016/j.bbi.2017.06.011, PMID: 28645775 PMC5650936

[23] Suh S Kim H Yang HC Cho ER Lee SK Shin C . Longitudinal course of depression scores with and without insomnia in non-depressed individuals: a 6-year follow-up longitudinal study in a Korean cohort. Sleep. (2013) 36:369–76. doi: 10.5665/sleep.2452, PMID: 23449814 PMC3571754

[24] Riemann D Voderholzer U . Primary insomnia: a risk factor to develop depression? J Affect Disord. (2003) 76:255–9. doi: 10.1016/s0165-0327(02)00072-1, PMID: 12943956

[25] Baglioni C Battagliese G Feige B Spiegelhalder K Nissen C Voderholzer U . Insomnia as a predictor of depression: a meta-analytic evaluation of longitudinal epidemiological studies. J Affect Disord. (2011) 135:10–9. doi: 10.1016/j.jad.2011.01.011, PMID: 21300408

[26] Tsuno N Besset A Ritchie K . Sleep and depression. J Clin Psychiatry. (2005) 66:1254–69. doi: 10.4088/jcp.v66n1008, PMID: 16259539

[27] Lee E Cho HJ Olmstead R Levin MJ Oxman MN Irwin MR . Persistent sleep disturbance: a risk factor for recurrent depression in community-dwelling older adults. Sleep. (2013) 36:1685–91. doi: 10.5665/sleep.3128, PMID: 24179302 PMC3792386

[28] Yan B Zhao B Jin X Xi W Yang J Yang L . Sleep efficiency may predict depression in a large population-based study. Front Psychiatry. (2022) 13:838907. doi: 10.3389/fpsyt.2022.838907, PMID: 35492719 PMC9043133

[29] Jiang J Li Z Li H Yang J Ma X Yan B . Sleep architecture and the incidence of depressive symptoms in middle-aged and older adults: A community-based study. J Affect Disord. (2024) 352:222–8. doi: 10.1016/j.jad.2024.02.020, PMID: 38342319

[30] Armitage R . Sleep and circadian rhythms in mood disorders. Acta Psychiatr Scand Suppl. (2007) 115:104–15. doi: 10.1111/j.1600-0447.2007.00968.x, PMID: 17280576

[31] Landsness EC Goldstein MR Peterson MJ Tononi G Benca RM . Antidepressant effects of selective slow wave sleep deprivation in major depression: a high-density EEG investigation. J Psychiatr Res. (2011) 45:1019–26. doi: 10.1016/j.jpsychires.2011.02.003, PMID: 21397252 PMC3119746

[32] Goldstein AN Greer SM Saletin JM Harvey AG Nitschke JB Walker MP . Tired and apprehensive: anxiety amplifies the impact of sleep loss on aversive brain anticipation. J Neurosci. (2013) 33:10607–15. doi: 10.1523/jneurosci.5578-12.2013, PMID: 23804084 PMC3693050

[33] Liston C Chen AC Zebley BD Drysdale AT Gordon R Leuchter B . Default mode network mechanisms of transcranial magnetic stimulation in depression. Biol Psychiatry. (2014) 76:517–26. doi: 10.1016/j.biopsych.2014.01.023, PMID: 24629537 PMC4209727

[34] Irwin MR . Why sleep is important for health: a psychoneuroimmunology perspective. Annu Rev Psychol. (2015) 66:143–72. doi: 10.1146/annurev-psych-010213-115205, PMID: 25061767 PMC4961463

[35] Zhao B Liu P Wei M Li Y Liu J Ma L . Chronic sleep restriction induces Aβ Accumulation by disrupting the balance of Aβ Production and clearance in rats. Neurochem Res. (2019) 44:859–73. doi: 10.1007/s11064-019-02719-2, PMID: 30632087

[36] Xie L Kang H Xu Q Chen MJ Liao Y Thiyagarajan M . Sleep drives metabolite clearance from the adult brain. Science. (2013) 342:373–7. doi: 10.1126/science.1241224, PMID: 24136970 PMC3880190

[37] Xin J Wang C Cheng X Xie C Zhang Q Ke Y . CX3C-chemokine receptor 1 modulates cognitive dysfunction induced by sleep deprivation. Chin Med J (Engl). (2021) 135:205–15. doi: 10.1097/cm9.0000000000001769, PMID: 34732662 PMC8769116

[38] Zhao Q Peng C Wu X Chen Y Wang C You Z . Maternal sleep deprivation inhibits hippocampal neurogenesis associated with inflammatory response in young offspring rats. Neurobiol Dis. (2014) 68:57–65. doi: 10.1016/j.nbd.2014.04.008, PMID: 24769004

[39] Irwin M McClintick J Costlow C Fortner M White J Gillin JC . Partial night sleep deprivation reduces natural killer and cellular immune responses in humans. FASEB J. (1996) 10:643–53. doi: 10.1096/fasebj.10.5.8621064, PMID: 8621064

[40] Yang TY Huang YH Cai HM Yu QW . Effects of complete and incomplete sleep deprivation on immune function of mice. Xi Bao Yu Fen Zi Mian Yi Xue Za Zhi. (2010) 26:115–7. doi: 10.13423/j.cnki.cjcmi.005367, PMID: 20230667

[41] Piber D Cho JH Lee O Lamkin DM Olmstead R Irwin MR . Sleep disturbance and activation of cellular and transcriptional mechanisms of inflammation in older adults. Brain Behav Immun. (2022) 106:67–75. doi: 10.1016/j.bbi.2022.08.004, PMID: 35953022

[42] Irwin MR . Insomnia and inflammation conspire to heighten depression risk: implications for treatment and prevention of mood disorders. Biol Psychiatry. (2025) 98:.819–829 doi: 10.1016/j.biopsych.2025.04.018, PMID: 40328368 PMC13528946

[43] Fan KQ Li YY Wang HL Mao XT Guo JX Wang F . Stress-induced metabolic disorder in peripheral CD4(+) T cells leads to anxiety-like behavior. Cell. (2019) 179:864–879.e19. doi: 10.1016/j.cell.2019.10.001, PMID: 31675497

[44] Yu X Ye L Liang H Li H Gao S Xu C . The alterations in CD4(+)Treg cells across various phases of major depression. J Affect Disord. (2024) 362:485–92. doi: 10.1016/j.jad.2024.07.037, PMID: 39009318

[45] Grosse L Hoogenboezem T Ambrée O Bellingrath S Jörgens S de Wit HJ . Deficiencies of the T and natural killer cell system in major depressive disorder: T regulatory cell defects are associated with inflammatory monocyte activation. Brain Behav Immun. (2016) 54:38–44. doi: 10.1016/j.bbi.2015.12.003, PMID: 26674997

[46] Chen J Wang X Cui T Ni Q Zhang Q Zou D . Th1-like Treg in vitiligo: An incompetent regulator in immune tolerance. J Autoimmun. (2022) 131:102859. doi: 10.1016/j.jaut.2022.102859, PMID: 35792518

[47] Jiang Z Wang H Wang X Duo H Tao Y Li J . TMED4 facilitates regulatory T cell suppressive function via ROS homeostasis in tumor and autoimmune mouse models. J Clin Invest. (2024) 135:e179874. doi: 10.1172/jci179874, PMID: 39480507 PMC11684806

[48] Filiano AJ Xu Y Tustison NJ Marsh RL Baker W Smirnov I . Unexpected role of interferon-γ in regulating neuronal connectivity and social behaviour. Nature. (2016) 535:425–9. doi: 10.1038/nature18626, PMID: 27409813 PMC4961620

[49] Bell JA Kivimäki M Bullmore ET Steptoe A Carvalho LA . Repeated exposure to systemic inflammation and risk of new depressive symptoms among older adults. Transl Psychiatry. (2017) 7:e1208. doi: 10.1038/tp.2017.155, PMID: 28809860 PMC5611724

[50] Wang F Zhu D Cao L Wang S Tong Y Xie F . Peripheral CD4(+) T helper lymphocytes alterations in major depressive disorder: A systematic review and meta-analysis. Neuroscience. (2024) 555:145–55. doi: 10.1016/j.neuroscience.2024.07.027, PMID: 39059741

[51] Kim J Suh YH Chang KA . Interleukin-17 induced by cumulative mild stress promoted depression-like behaviors in young adult mice. Mol Brain. (2021) 14:11. doi: 10.1186/s13041-020-00726-x, PMID: 33441182 PMC7805143

[52] Backhaus J Junghanns K Broocks A Riemann D Hohagen F . Test-retest reliability and validity of the Pittsburgh Sleep Quality Index in primary insomnia. J Psychosom Res. (2002) 53:737–40. doi: 10.1016/s0022-3999(02)00330-6, PMID: 12217446

[53] Irwin MR Olmstead R Carrillo C Sadeghi N Nicassio P Ganz PA . Tai chi chih compared with cognitive behavioral therapy for the treatment of insomnia in survivors of breast cancer: A randomized, partially blinded, noninferiority trial. J Clin Oncol. (2017) 35:2656–65. doi: 10.1200/jco.2016.71.0285, PMID: 28489508 PMC5549450

[54] Spiegelhalder K Regen W Nissen C Feige B Baglioni C Riemann D . Magnetic resonance spectroscopy in patients with insomnia: A repeated measurement study. PloS One. (2016) 11:e0156771. doi: 10.1371/journal.pone.0156771, PMID: 27285311 PMC4902218

[55] Kamal F Morrison C Dadar M . Investigating the relationship between sleep disturbances and white matter hyperintensities in older adults on the Alzheimer’s disease spectrum. Alzheimers Dement (Amst). (2024) 16:e12553. doi: 10.1002/dad2.12553, PMID: 38476639 PMC10927930

[56] Pigeon WR Britton PC Ilgen MA Chapman B Conner KR . Sleep disturbance preceding suicide among veterans. Am J Public Health. (2012) 102:S93–7. doi: 10.2105/ajph.2011.300470, PMID: 22390611 PMC3496468

[57] Zheng Y Li Y Cai H Kou W Yang C Li S . Alterations of peripheral lymphocyte subsets in isolated rapid eye movement sleep behavior disorder. Mov Disord. (2024) 39:1179–89. doi: 10.1002/mds.29798, PMID: 38529776

[58] Tasali E Leproult R Spiegel K . Reduced sleep duration or quality: relationships with insulin resistance and type 2 diabetes. Prog Cardiovasc Dis. (2009) 51:381–91. doi: 10.1016/j.pcad.2008.10.002, PMID: 19249444

[59] Cohen S Doyle WJ Alper CM Janicki-Deverts D Turner RB . Sleep habits and susceptibility to the common cold. Arch Intern Med. (2009) 169:62–7. doi: 10.1001/archinternmed.2008.505, PMID: 19139325 PMC2629403

[60] Tobaldini E Costantino G Solbiati M Cogliati C Kara T Nobili L . Sleep, sleep deprivation, autonomic nervous system and cardiovascular diseases. Neurosci Biobehav Rev. (2017) 74:321–9. doi: 10.1016/j.neubiorev.2016.07.004, PMID: 27397854

[61] van Leeuwen WM Lehto M Karisola P Lindholm H Luukkonen R Sallinen M . Sleep restriction increases the risk of developing cardiovascular diseases by augmenting proinflammatory responses through IL-17 and CRP. PloS One. (2009) 4:e4589. doi: 10.1371/journal.pone.0004589, PMID: 19240794 PMC2643002

[62] Kolstad HA . Nightshift work and risk of breast cancer and other cancers–a critical review of the epidemiologic evidence. Scand J Work Environ Health. (2008) 34:5–22. doi: 10.5271/sjweh.1194, PMID: 18427694

[63] Ferrie JE Shipley MJ Cappuccio FP Brunner E Miller MA Kumari M . A prospective study of change in sleep duration: associations with mortality in the Whitehall II cohort. Sleep. (2007) 30:1659–66. doi: 10.1093/sleep/30.12.1659, PMID: 18246975 PMC2276139

[64] Bryant PA Trinder J Curtis N . Sick and tired: Does sleep have a vital role in the immune system? Nat Rev Immunol. (2004) 4:457–67. doi: 10.1038/nri1369, PMID: 15173834

[65] Besedovsky L Lange T Haack M . The sleep-immune crosstalk in health and disease. Physiol Rev. (2019) 99:1325–80. doi: 10.1152/physrev.00010.2018, PMID: 30920354 PMC6689741

[66] Kruse JL Congdon E Olmstead R Njau S Breen EC Narr KL . Inflammation and improvement of depression following electroconvulsive therapy in treatment-resistant depression. J Clin Psychiatry. (2018) 79:9042. doi: 10.4088/JCP.17m11597, PMID: 29489077 PMC6013272

[67] Miller AH Felger JC Haroon E . Designing clinical trials for an inflammatory subtype of major depressive disorder. Biol Psychiatry. (2025). doi: 10.1016/j.biopsych.2025.04.003, PMID: 40216052 PMC13425400

